# Supplementary material for: Evaluating the antibody response to SARS-COV-2 vaccination amongst kidney transplant recipients at a single nephrology centre
Source: PLoS One. 2022 Mar 10;17(3):e0265130. doi: 10.1371/journal.pone.0265130 (PMC8912185; doi:10.1371/journal.pone.0265130)
Supplement: Attachment — Submitted filename: plos one review.docx [file pone.0265130.s002.docx]

This paper demonstrates poor seroconversion following two doses of either a Pfizer or AstraZeneca SARS-COV-2 vaccine in kidney transplant recipients and the effect mycophenolate, older age and eGFR. Although these are significant findings very similar findings have been reported previously in large cohorts from America, Israel, France and the UK. This is one of the first reports to include the AstraZeneca vaccine although its use was not found to be predictive of seroconversion or seronegativity. The authors claims are properly placed in the context of previous literature. The data and analyses support the claims. There are a few bits of data that I believe need to be clarified. The methods suggest that when the exact date of vaccination was unavailable the date was estimated from the date from the first vaccination. The methods also state to patients had to have a their antibody test at least 8 days from the date of second vaccination. I think it’s important to know how many patients have an estimated date versus an actual date as that may have a significant impact on the results especially given the changes in JCVI recommended timing. The serological testing needs to specify what the antibody was against as we know this has an impact in transplant patients. In this case, the assay used was against the S1 RBD, which appears to perform best in transplant patients. This should be included. The analyses consisting of a univariate analysis, a multivariate analysis and a propensity score analysis appear appropriate. The manuscript is well organised and written clearly.

Some particular points

2.34: use SAR-COV-2 rather than SARS. Repeated again 1.36 twice

2.44 older age

2.68-71 I am unsure if reporting the reported ranges and the pooled seroconversion rates is helpful especially without mentioning it is a pooled analysis of the reported ranges

4.86-94 and 5.97 some repetition here in the study group and vaccines used

5.98 8 days seems a short interval to assess antibody response. I notice the mean was considerably longer

6.131 as above how many patients had estimated vaccination dates as opposed to known vaccination dates

7.145 mention which antibodies were measured

Discussion:

Might be worth mentioning the likely confounding with BK viremia and reduced immunosuppression . Interesting discussion re the potential reasons for higher seroprevalence including the longer time since transplant and the longer time between vaccines. Discussion on the impact the different vaccines on seroconversion could reference the other recently published UK paper that compares AZ and Pfizer vaccines in kidney transplant recipients.
