# Supplementary material for: Evaluating the antibody response to SARS-COV-2 vaccination amongst kidney transplant recipients at a single nephrology centre
Source: PLoS One. 2022 Mar 10;17(3):e0265130. doi: 10.1371/journal.pone.0265130 (PMC8912185; doi:10.1371/journal.pone.0265130)
Supplement: Attachment — Submitted filename: Response to reviewers.docx [file pone.0265130.s003.docx]

**Dear Reviewers,**

Thank you for your excellent review of this manuscript. Your comments were very insightful. Please find below our response to your comments and the changes we have made to the manuscript as a result. (highlighted in yellow)

**Comments to the Author**

Review Comments to the Author)

**Reviewer #1:** Congratulations on writing this paper. This paper demonstrates poor seroconversion following two doses of either a Pfizer or AstraZeneca SARS-COV-2 vaccine in kidney transplant recipients and the effect mycophenolate, older age and eGFR. Although these are significant findings very similar findings have been reported previously in large cohorts from America, Israel, France and the UK. This is one of the first reports to include the AstraZeneca vaccine although its use was not found to be predictive of seroconversion or seronegativity. The authors claims are properly placed in the context of previous literature. The data and analyses support the claims. **There are a few bits of data that I believe need to be clarified**. The methods suggest that when the exact date of vaccination was unavailable the date was estimated from the date from the first vaccination. The methods also state to patients had to have their antibody test at least 8 days from the date of second vaccination. I think it’s important to know how many patients have an estimated date versus an actual date as that may have a significant impact on the results especially given the changes in JCVI recommended timing.

**Response**: Thanks for your comment. Thirteen subjects (13/373) had their vaccine dosing interval estimated using the operational JCVI dosing interval at the time of vaccination. This has been included in the manuscript

The serological testing needs to specify what the antibody was against as we know this has an impact in transplant patients. In this case, the assay used was against the S1 RBD, which appears to perform best in transplant patients. This should be included. The analyses consisting of a univariate analysis, a multivariate analysis and a propensity score analysis appear appropriate. The manuscript is well organised and written clearly.

Some particular points

2.34: use SAR-COV-2 rather than SARS. Repeated again 1.36 twice

**Response**: Thank you for pointing this out. It has been corrected.

2.44 older age

**Response**: Thank you for pointing this out. It has been changed to ‘increasing age’

2.68-71 I am unsure if reporting the reported ranges and the pooled seroconversion rates is helpful especially without mentioning it is a pooled analysis of the reported ranges

**Response**: Thank you very much I have removed this from the manuscript

4.86-94 and 5.97 some repetition here in the study group and vaccines used

**Response**: Thank you We have removed the repetition of the vaccine names

5.98 8 days seems a short interval to assess antibody response. I notice the mean was considerably longer.

**Response**: Thank you for the observation. We chose 8 days because evidence suggests that antibodies to SARS-COV 2 appear within 1-3 weeks following vaccination. This may however be longer in immunocompromised patients. However, as you rightly pointed out, the median (IQR) time to antibody assay was 38(22-55) while the mean (SD) time to antibody assay was 42(23) days (We will add this to the limitations of our study.

6.131 as above how many patients had estimated vaccination dates as opposed to known vaccination dates:

**Response**: Thank you. As highlighted above, thirteen subjects (13/373) had their vaccine dosing interval estimated using the operational JCVI dosing interval at the time of vaccination.

7.145 mention which antibodies were measured

**Response**: Thank you, this has been added to the manuscript

Discussion:

Might be worth mentioning the likely confounding with BK viremia and reduced immunosuppression. Interesting discussion re the potential reasons for higher seroprevalence including the longer time since transplant and the longer time between vaccines. Discussion on the impact the different vaccines on seroconversion could reference the other recently published UK paper that compares AZ and Pfizer vaccines in kidney transplant recipients.

**Response**: Thank you, I have added a few lines on the likely confounding effect of history of BK viremia in the result. I have also included the recent study that compared AZD vs Pfizer vaccines

**Reviewer #2**: Chukwu et al presented a well-written paper on antibody response in kidney transplant recipients to the COVID-19 vaccine. Their retrospective analysis was clearly conveyed, and they were transparent in their analysis. While the authors make a great point about the lack of clinical trials studying the COVID-19 vaccine in transplant recipients, their findings are not novel and only contribute to the multitude of papers reporting on the same thing. While there is value in adding to the body of literature on this topic, there should be a more novel angle to their findings as their PSA also did not find anything different. More pertinent studies, such as third vaccine dosing or HLA changes due to antibody responses or revaccination antibody response following transplantation, may have more clinical weight for the readers.

**Response**: Thank you for an excellent review, I hope the changes I have made in response to your review is satisfactory. We are currently in the process of publishing the results of the impact of the 3^rd^ and 4^th^ vaccine doses on antibody response and we feel this will be better as a standalone paper rather than adding it to the current paper. We feel this paper does add to the body of evidence supporting the lower immune response to COVID vaccines by transplant patients. We believe that our use of propensity score analysis to reduce the effect of a broader range of confounders on important outcomes provided an additional validity to the study

Ten more references have been added to the reference list in response the reviewers’ requirements with regards to impact of the different vaccine types on immune response, effect of a previous history of BK viremia on antibody response and the details of the antibody assay used.
